# Landscape and predictions of inflammatory bowel disease in China: China will enter the Compounding Prevalence stage around 2030

**DOI:** 10.3389/fpubh.2022.1032679

**Published:** 2022-10-25

**Authors:** Bule Shao, Wenjing Yang, Qian Cao

**Affiliations:** ^1^Department of Gastroenterology, Sir Run Run Shaw Hospital, Zhejiang University School of Medicine, Hangzhou, China; ^2^Zhejiang University-University of Edinburgh Institute, Zhejiang University School of Medicine, Hangzhou, China

**Keywords:** disease burden, China, prediction, public health, inflammatory bowel disease, age-period-cohort analysis

## Abstract

**Background:**

This study aims to explore the epidemiological trends of inflammatory bowel disease (IBD) over the past three decades in China and further predict the trends of IBD in the next 25 years.

**Methods:**

The prevalence, incidence, mortality, years of life lived with disability (YLDs), years of life lost (YLLs), disability-adjusted life years (DALYs), and annual percentage changes of the above metrics of IBD in China from 1990 to 2019 were extracted from the Global Burden of Disease Study 2019. The corresponding trends in the next 25 years were predicted.

**Results:**

From 1990 to 2019, the cases of IBD in China raised to 484 thousand [95% uncertainty interval (UI) 411–571] and 427 thousand (366–498) among males and females, respectively. The age-standardized incidence rate of IBD increased from 1.72 per 100,000 population (1.44–2.05) to 3.35 per 100,000 population (2.88–3.88) among males and from 1.20 per 100,000 population (1.02–1.42) to 2.65 per 100,000 population (2.29–3.08) among females. The highest incidence rate occurred in people aged 35–39 years. The total YLDs attributed to IBD significantly increased, but the YLLs showed a decreasing trend, resulting in minor alterations of the DALYs. In the next 25 years, the incidence of IBD would continue to increase until a plateau by 2030, and IBD-related deaths would also increase to about 7.57 thousand by 2044 despite the decreasing age-standardized mortality rate. Similar trends were observed for both sexes, with a slight male predominance.

**Conclusions:**

Although China is still a low-endemic area of IBD, the prevalence and incidence of IBD dramatically increased in the past three decades. The burden of IBD in China is expected to grow continuously in the next 25 years due to the large population base and severe aging problem. China is estimated to enter the Compounding Prevalence stage around 2030.

## Introduction

Inflammatory bowel disease (IBD), including Crohn's disease and ulcerative colitis, is a chronic inflammatory condition of the gastrointestinal tract that affects more than 6.8 million people worldwide (1). Although the cause is incompletely understood, IBD is suggested to result from a dysregulated immune response against luminal and microbial antigens in a genetically susceptible individual encountering environmental triggers such as dietary changes and antibiotic use (2). As two distinct pathophysiological entities, Crohn's disease can affect any segments of the gastrointestinal tract in an asymmetrical and transmural manner, while ulcerative colitis is limited to the colonic mucosa (3, 4). Common symptoms of IBD include abdominal pain and chronic diarrhea. Crohn's disease is often associated with weight loss and perianal diseases, while rectal bleeding occurs more frequently in ulcerative colitis (5). Besides, about 30–50% of patients with IBD suffer from extraintestinal manifestations that typically affect the eyes, skin, liver, and joints (5). Most patients are diagnosed in adolescence and early adulthood, the most productive time of life (5). With an unpredictable relapsing and remitting course, IBD adversely impacts all aspects of life in patients, leading to substantial social costs that include both health-care-related costs and indirect costs such as absenteeism and early retirement (6).

Histologically, IBD has been considered a disease in western countries, but epidemiological studies over the last two decades have reported a shift, suggesting that IBD incidence has reached a plateau in high-income countries while rapidly rising in newly industrialized countries such as China (7). China is one of the largest developing countries, accounting for up to 20% of the world's population. In China, the first case of ulcerative colitis was reported in 1956, and the diagnostic criteria of ulcerative colitis were published firstly in 1973, before which the disease was not well-recognized (8). Reports of Crohn's disease were even lagging, mainly due to the complicated and non-specific clinical manifestations (9). Since Chinese gastroenterologists were unfamiliar with IBD, the prevalence and incidence of IBD remained low in China for a long time. Unlike the abundant epidemiological data in western countries, few studies have reported the burden of IBD in China. In the absence of a nationwide registration system, most of the prospective studies are sparse and only report IBD incidence in several regions of China during a short time period (9). Thus, it is necessary to conduct a systemic long-term assessment of the national burden of IBD in China.

Launched in 1991, the Global Burden of Disease Study (GBD) has continuously developed and updated a comprehensive and internally consistent database for modeling the global burden of diseases, injuries, and risk factors (10). Based on the latest GBD 2019 estimates in terms of prevalence, incidence, mortality, years lived with disability (YLDs), years of life lost (YLLs), and disability-adjusted life-years (DALYs), this study evaluates the disease burden and temporal trends of IBD in China. To get a deeper insight into future trends, we also predict the number and rates of IBD incidence and mortality in the next 25 years (until 2044). Our results provide an updated overview of the IBD burden in China, which can be crucial for policymakers to develop relevant strategies to cope with the burden of this socially and economically costly disease.

## Methods

### Overview

The GBD study provides a standardized approach for estimating prevalence, incidence, deaths, YLLs, YLDs, and DALYs—for 23 age groups; males, females, and both sexes combined; and 204 countries and territories that are grouped into 21 regions and seven super-regions.

The GBD 2019 used multiple methods to integrate a large number of available data sources with the specific epidemiology of each disease, and the Bayesian meta-regression tool DisMod-MR 2.1 was used as the main method of estimation (11). Detailed information about data resources, definitions, statistical modeling, and efforts to improve data quality has been previously reported (10, 11).

We extracted GBD 2019 data on IBD in China from 1990 to 2019 using the Global Health Data Exchange (GHDx) (12). The variables obtained from these data included year, age, sex, and location. The incidence, prevalence, mortality, and DALYs were used as the primary metrics to assess the impacts on health within the population. The total number of IBD cases and rates of each metric was provided by the GBD 2019. Prevalence rate (per 100,000) was defined as aggregated cases (including new cases and previously diagnosed cases) divided by the population size; incidence rate (per 100,000) was defined as the number of new cases divided by the population size; mortality rate (per 100,000) was defined as the number of annual deaths divided by the total population size; YLLs were calculated as the sum of each death multiplied by the standard life expectancy at each age and DALYs were summed by YLLs and YLDs.

For the prediction of the IBD burden, the predicted Chinese population was obtained from the United Nations World Population Prospects 2019 Revision by year, sex, and age (https://population.un.org/wpp/Download/Standard/Population/).

The Institutional Review Board of Sir Run Run Shaw Hospital of Zhejiang University determined that the study did not need approval since the data used were publicly available. This study complied with the Guidelines for Accurate and Transparent Health Estimates Reporting (GATHER) recommendations (13).

### Statistical analysis

All rates are expressed as age-standardized based on the GBD reference population unless otherwise specified. Uncertainty is estimated with each metric and propagated throughout the GBD modeling process. 95% uncertainty intervals (95% UIs) are calculated by the 2.5th and 97.5th percentiles of the draw-level values (1,000 draws for each metric) (11). The annual percentage change was extracted from GHDx to evaluate trends in disease burden (12).

The package nordpred in the open-source R program was developed by the Norwegian cancer registry, which has been shown to perform well in predicting the incidence and mortality trend of cancer and chronic disease (14, 15). The nordpred was used to perform an age-period-cohort (APC) analysis to predict the numbers and rates of the incidence and mortality of IBD in the next 25 years, taking into account both the change in rates and the population structure. The nordpred package can apply the power5 and Poisson APC models to perform the prediction (https://github.com/haraldwf/nordpred). As Møller et al. (16) recommended, the power5 function, instead of the logarithm function, was used as a link function to level off the exponential growth. Analyses were conducted using the R program (Version 4.0.1; R core team, R Foundation for Statistical Computing, Vienna, Austria). The ggplot2 packages of the R program were used to perform the visualization of the results (17).

## Results

### Temporal trends of the burden metrics of IBD in China from 1990 to 2019

**Figure 1:**
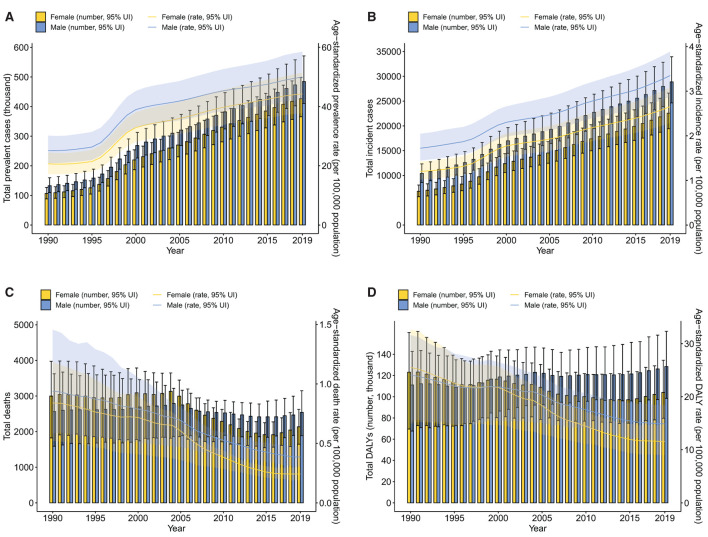
Trends from 1990 to 2019 in number and age-standardized rates of prevalence **(A)**, incidence **(B)**, deaths **(C)**, and DALYs **(D)** of IBD by sex in China. Error bars indicate the 95% uncertainty interval (UI) for the number of each measure. Shading indicates the 95% UI for the age-standardized rates. DALYs, disability-adjusted life-years; IBD, inflammatory bowel disease.

Inflammatory bowel disease prevalence significantly increased from 1990 to 2019 among the Chinese population (Figure 1A; Supplementary Table 1). The number of prevalent cases increased by about four times among females, from 107 thousand (95% UI 88.2–127) in 1990 to 427 thousand (95% UI 366–498) in 2019. A similar increase was observed among males, from 133 thousand (95% UI 110–160) to 484 thousand (95% UI 411–571). The age-standardized prevalence rate increased from 20.7 per 100,000 population (95% UI 17.2–24.5) in 1990 to 44.2 per 100,000 population (95% UI 37.7–51.4) in 2019 among females, with an annual change rate of 1.14% (95% CI 1.05–1.26). For the male population, the age-standardized prevalence rate showed an annual percentage change of 0.99% (95% CI 0.90–1.09) between 1990 and 2019, from 25.1 per 100,000 population (95% UI 20.9–30.2) to 50.0 per 100,000 population (95% UI 42.5–58.5). Both the number of prevalent cases and age-standardized prevalence rate were higher in males than females in all years from 1990 to 2019.

From 1990 to 2019, the number of incident cases of IBD increased by about three times in China, from 6.81 thousand (95% UI 5.70–8.07) to 22.6 thousand (95% UI 19.4–26.6) among females while from 10.4 thousand (95% UI 8.59–12.3) to 28.9 thousand (95% UI 24.6–33.9) among males (Figure 1B; Supplementary Table 1). In terms of the age-standardized incidence rate, the female population showed an annual change rate of 1.20% (95% CI 1.12–1.31) between 1990 and 2019, from 1.20 per 100,000 population (95% UI 1.02–1.42) to 2.65 per 100,000 population (95% UI 2.29–3.08), while the male population presented with an annual change rate of 0.95% (95% CI 0.86–1.04), increasing from 1.72 per 100,000 population (95% UI 1.44–2.05) in 1990 to 3.35 per 100,000 population (95% UI 2.88–3.88) in 2019. Similar to the prevalence, both the number of incident cases and age-standardized incidence rate were significantly higher in males in all the years assessed.

Although the prevalence and incidence of IBD have been increasing steadily in China in the past three decades, mortality associated with IBD generally showed a decreasing trend (Figure 1C; Supplementary Table 1). The trough of the total number of IBD-related deaths occurred in 2015 among both females and males. Nevertheless, the number of deaths among females decreased from 2,999 (95% UI 1,825–3,974) in 1990 to 2,135 (95% UI 1,647–2,625) in 2019 while the number of deaths among males showed no significant decrease [2,561 (95% UI 1,591–3,625) in 1990 to 2,541 (95% UI 1,885–3,148) in 2019]. However, the age-standardized death rate declined over time for both sexes. Specifically, from 1990 to 2019, the age-standardized death rate decreased from 0.84 per 100,000 population (95% UI 0.52–1.18) to 0.24 per 100,000 population (95% UI 0.19–0.30) among females, with an annual change rate of −0.71% (95% CI: −0.81, −0.51) while among males, decreasing from 0.94 per 100,000 population (95% UI 0.58–1.46) to 0.38 per 100,000 population (95% UI 0.30–0.47), with an annual change rate of −0.59% (95% CI: −0.76, −0.35).

Disability-adjusted life years attributed to IBD, comprising YLDs and YLLs, generally decreased among both females and males in China from 1990 to 2019 (Figure 1D; Supplementary Table 1). However, between 1990 and 2019, the total YLDs increased by more than three times for both sexes. Tendencies in the age-standardized YLD rate were similar between females and males over the study period with an annual change rate of 1.08% (95% CI 0.96–1.24) and 0.98% (95% CI 0.84–1.13) for females and males, respectively (Supplementary Figure S1A; Supplementary Table 1). Different from YLDs, decreasing trends were observed in both the total YLLs attributed to IBD and the age-standardized YLL rates among both sexes from 1990 to 2019, with a slower decreasing rate in males than females (Supplementary Figure S1B). In terms of the age-standardized YLL rate, annual change rates between 1990 and 2019 were −0.79% (95% CI: −0.85, −0.56) and −0.63% (95% CI: −0.75, −0.35) for females and males, respectively (Supplementary Table 1). The total DALYs from 1990 to 2019 fluctuated among females, slightly decreasing from 123 thousand (95% UI 69.6–161) to 104 thousand (95% UI 79.7–134), while the total DALYs among males increased from 111 thousand (95% UI 67.6–143) in 1990 to 128 thousand (95% UI 98.5–162) in 2019. However, the age-standardized DALY rates from 1990 to 2019 consistently decreased, from 25.5 per 100,000 population (95% UI 14.9–32.6) to 11.6 per 100,000 population (95% UI 8.99–14.8) among females while from 24.1 per 100,000 population (95% UI 15.4–31.6) to 14.9 per 100,000 population (95% UI 11.5–18.5) among males. Corresponding annual change rates were −0.55% (95% CI: −0.67, −0.18) and −0.38% (95% CI: −0.56, −0.05) for females and males, respectively.

### Age patterns of the burden metrics of IBD in China in 2019

**Figure 2:**
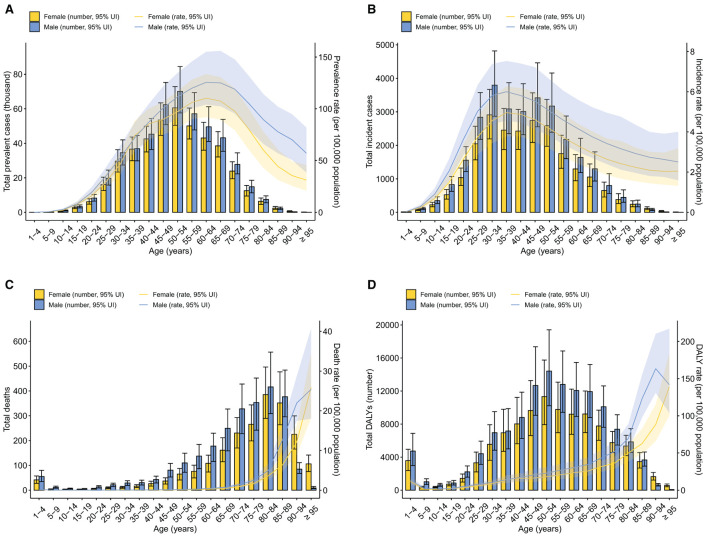
Age patterns by sex in 2019 of number and age-specific rates of prevalence **(A)**, incidence **(B)**, deaths **(C)**, and DALYs **(D)** due to IBD in China. Error bars indicate the 95% uncertainty interval (UI) for the number of each measure. Shading indicates the 95% UI for the age-standardized rates. DALYs, disability-adjusted life-years; IBD, inflammatory bowel disease.

The burden of IBD in 2019 showed distinct age patterns among the Chinese population (Figure 2; Supplementary Tables 2, 3). The number of individuals with IBD was highest in the group aged 50–54 years for both sexes [60.6 thousand (95% UI 50.4–72.8) in females and 70.1 thousand (95% UI 57.6–84.5) in males] (Figure 2A). The IBD age-specific prevalence rate peaked at age 60–64 years among both females and males and was higher in males than females among those over 40. The prevalence rate was over 100 per 100,000 population among females aged between 55 and 69 years and among males aged between 45 and 79 years. The number of incident cases in 2019 was over 2,000 among females aged between 25 and 54 years and males aged between 25 and 59 years (Figure 2B). The age-specific incidence rate peaked in the 35–39 year age group among both females and males [4.96 per 100,000 population (95% UI 3.78–6.26) in females and 6.00 per 100,000 population (95% UI 4.57–7.53) in males] and was consistently higher in males than females. In 2019, the highest number of IBD-related deaths occurred in the group aged 80–84 years among both females and males [385 (95% UI 289–496) in females and 416 (95% UI 309–557) in males], whereas the age-specific death rate began to increase rapidly from the age of 60 and was highest in the group aged 95 years and older among both females and males [25.9 per 100,000 population (95% UI 18.3–34.5) in females and 25.4 per 100,000 population (95% UI 18.0–40.7) in males] (Figure 2C).

The total YLDs attributed to IBD reached a peak in the group aged 50–54 years among both females and males, whereas the age-specific YLD rate was highest among those aged 60–64 years [15.8 per 100,000 population (95% UI 10.1–23.0) in females and 18.0 per 100,000 population (95% UI 11.5–26.2) in males] (Supplementary Figure S1C). The total YLLs were highest among males aged 70–74 years [6.26 thousand (95% UI 4.35–8.16)] and close to or over 4,000 among females aged 70–84 years (Supplementary Figure S1D). Years of life lost were higher in males than females before the age of 90. Notably, the YLLs of the 1–4 year age group were the fifth highest among females and the sixth highest among males. It is not surprising because each death at a young age will lead to more YLLs than at older ages. The age-specific YLL rate generally increased with age and peaked among males aged 90–94 years [154 per 100,000 population (95% UI 122–201)] and among females aged 95 years and older [134 per 100,000 population (95% UI 94.8–179)]. The total DALYs were above 8,000 among females between 40 and 69 years and males aged between 40 and 74 years (Figure 2D). The age-specific DALY rate generally increased with age and was highest among males aged 90–94 years [163 per 100,000 population (95% UI 131–210)] and among females aged over 95 years [139 per 100,000 population (95% UI 101–184)].

### Predictions of incidence and mortality of IBD in China from 2020 to 2044

**Figure 3:**
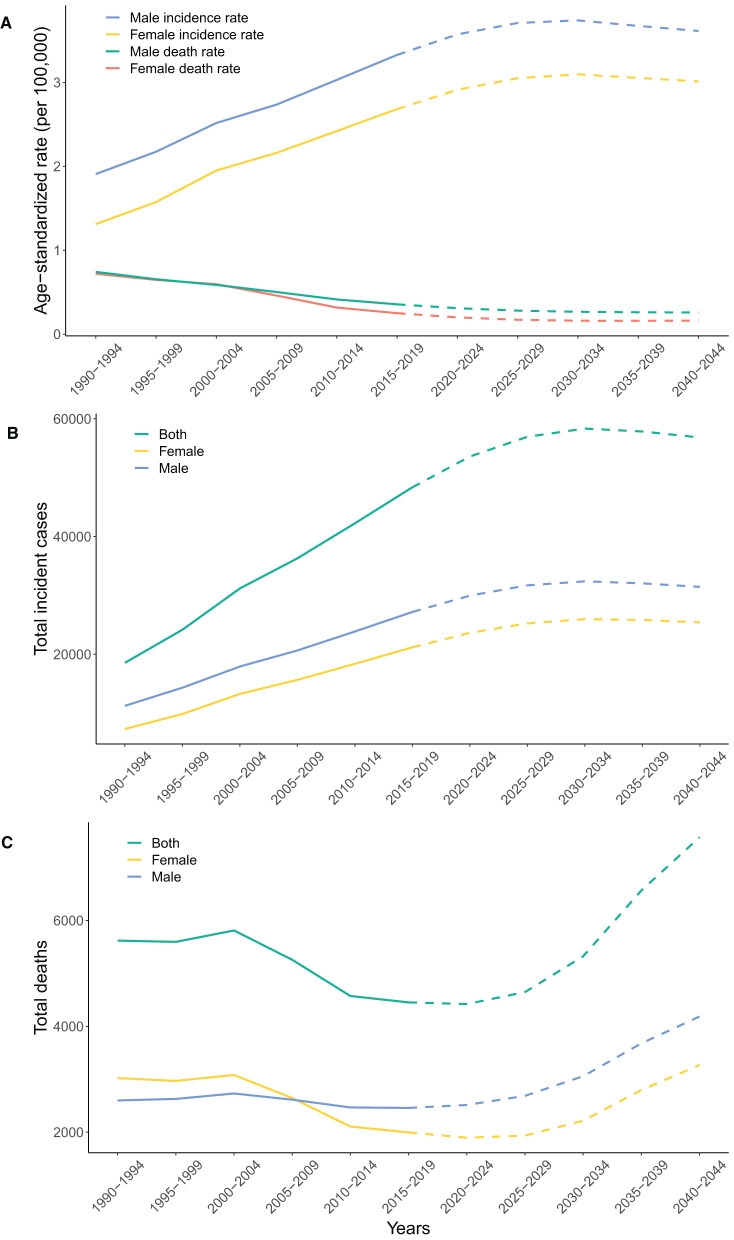
Observed trends from 1990 to 2019 (solid line) and predictions from 2020 to 2044 (dashed line) in age-standardized rates of incidence and deaths **(A)**, and the number of incident cases **(B)** and deaths **(C)** of IBD by sex in China. IBD, inflammatory bowel disease.

Based on GBD data on IBD in China from 1990 to 2019, we further predicted IBD incidence and related deaths and the corresponding rates in the next 25 years (Figure 3). The age-standardized incidence rates would show an upward trend but stabilize or even slightly decrease after 2030–2034 among both females and males (3.10 per 100,000 population in females and 3.74 per 100,000 population in males) (Figure 3A). However, the age-standardized death rates would gently decrease to a plateau by 2030 for both sexes (Figure 3A). The number of incident cases of IBD would increase until 2030–2034 (26.0 thousand in females and 32.4 thousand in males), after which IBD incidence would enter a plateau or even slightly decrease (Figure 3B). Inflammatory bowel disease-related deaths for both sexes would increase steadily from 2020 to 2044 to 7.57 thousand in 2040–2044, including 3.27 thousand females and 4.19 thousand males (Figure 3C). In the next 25 years, the age-standardized rates of incidence and deaths, together with the number of incident cases and deaths, would always be higher among males than among females.

## Discussion

China is one of the largest newly-industrialized countries, with the burden of IBD rising (9). Using the latest data from the GBD 2019, we comprehensively described the disease burden due to IBD in China by sex and age groups. Our results revealed that the prevalence, incidence, and YLDs of IBD continuously increased from 1990 to 2019 while the mortality, YLLs, and DALYs due to IBD generally showed a downward trend. We reported that approximately 900 thousand individuals were living with IBD in China in 2019, and the number of prevalent cases would increase steadily in the following years. To the best of our knowledge, this is the first study that predicted the numbers and rates of IBD incidence and mortality in China in the next 25 years. Very recently, Zhang et al. (18) updated the long-term trends of IBD burdens in China based on the same database GBD 2019. However, they did not provide any evaluation of future trends, which is critical for policy making. Our study highlights that the burden of IBD in China is expected to climb continuously in the next 25 years due to the large population base and fast-growing aging populations. The incident cases of IBD would reach nearly 60 thousand by 2030, and the IBD-related deaths would increase to about 7.57 thousand in 2044 despite a consistently low mortality rate. These findings indicate that China will transition to the third epidemiological stage, the Compounding Prevalence stage, around 2030. Thus, Chinese healthcare delivery systems and economies need to prepare for the ever-increasing burden of this socioeconomically costly disease.

The age-standardized incidence rate of IBD dramatically increased among the Chinese population from 1990 to 2019. Although the rise of IBD incidence is conserved in the literature, incidence rates in this study are higher than those estimated by previous systemic reviews (1.74/100,000 or 1.80/100,000), probably due to earlier reporting years, hospital-based data collection, and different estimation models in these reviews (19, 20). Similar trends have been observed in many other newly industrialized countries in Asia, South America, Eastern Europe, and Africa at the turn of the twenty-first century (7). A four-decade analysis of the IBD incidence in Malaysia documented a rapid rise from the 1980s (0.36 per 100,000 person-years) to the 2010s (1.46 per 100,000 person-years), especially in the past decade (21). Moreover, an epidemiological study in Seoul, Korea, during the period 1986–2015 demonstrated that by 5-year intervals, the mean annual incidence rate of IBD significantly increased from 0.06/100,000 to 2.44/100,000 for Crohn's disease and from 0.29/100,000 to 5.82/100,000 for ulcerative colitis (22). The incidence of IBD experienced a similar increase in the state of São Paulo, Brazil, from 1.0/100,000 in 1986–1990 to 13.3/100,000 in 2012–2015 (23, 24). Accelerating incidence rates of IBD in newly industrialized countries can be explained by two fundamental factors. Firstly, the rapid industrialization and urbanization in China have exposed genetically susceptible individuals to westernized environmental factors that might alter their intestinal microbiota (25). These risk factors include improved hygiene status, air pollution, greater exposure to antibiotics, diets containing high levels of fat and refined sugars, and other lifestyle changes such as cigarette smoking and discontinued breastfeeding (26). Secondly, economic advances contribute to better access to health care systems, more widely available diagnostic tools, increased awareness of both patients and physicians, and improved disease surveillance, all of which drive a higher diagnosis rate (6, 27).

The fatal burden of IBD (mortality and YLLs) attenuated in China from 1990 to 2019 and remained relatively low, whereas the non-fatal burden (YLDs) increased steadily. The overall decrease in mortality reflects the improved survival of patients with IBD in China, which can be attributed to the establishment of consensus guidelines for IBD management, increasing education of IBD specialists, earlier introduction of biological therapy, improvements in surgical techniques, and raised awareness of colorectal cancer surveillance (27, 28). Moreover, with the consolidation of the health insurance schemas in China, individuals become financially protected and get increasingly equitable access to health care (29). For example, the most widely used biologics, infliximab, became available under Chinese medical insurance schemes in 2020, helping to reduce the economic burden for patients with IBD (27). Despite recent progress in therapeutic approaches, IBD is still an incurable lifelong condition that can greatly compromise both the physical and psychological dimensions of life. Patients with IBD might suffer from symptoms of common mental disorders because of gut–brain interactions, chronicity of symptoms, and impaired quality of life (30). According to the most recent meta-analysis, up to a third of IBD patients are affected by anxiety, and the prevalence of depression in IBD patients is approximately 25%, much higher than that in the general population (3.4%) (31). However, only IBD-specific symptoms account for disability weights in GBD studies but not these psychological comorbidities or other accompanying inflammatory conditions (11).

Trends of all the burden metrics assessed are similar between males and females, but a slight male predominance is observed among patients with IBD in China, which is consistent with the meta-analysis performed by Li et al. (19). In western countries, most epidemiological studies have shown female predominance for adult Crohn's disease while data on the gender distribution of ulcerative colitis are less consistent (32). However, the male predominance for Crohn's disease and ulcerative colitis has been consistently reported among the Asian population (33). A retrospective study of a Taiwanese IBD cohort revealed that 71.1% of all the patients admitted between 2000 and 2018 were male (34). Sex-based epidemiology of IBD might be explained by the difference in environmental exposures. Smoking is one of the most well-established risk factors for IBD. The Global Adult Tobacco Survey has demonstrated that in China, the prevalence of current smoking in men (52.9%) is much higher than that in women (2.4%) (35). Generally, current smoking contributes to an increased risk of Crohn's disease but seemingly protects individuals from developing ulcerative colitis (36). However, IBD susceptibility attributed to smoking shows gender and ethnic variations, indicating complex gene–environment interactions (37). Besides the smoking hypothesis, some studies postulate that sex hormones might be responsible for IBD gender distributions by influencing the brain–gut–microbial axis, but detailed mechanisms remain unclear (38).

In our study, the age-specific incidence rate of IBD peaked at the age of 35–39 among the Chinese population in 2019. The long disease duration of IBD resulted in death rates that began to increase at the age of 60 and then peaked at the age of over 90. Thus, the highest prevalence rate occurred in people aged 60–64. According to other systemic reviews, the peak age at diagnosis is the 20s and 30s for Crohn's disease and ulcerative colitis, respectively (39). Some epidemiological studies have also reported a bimodal distribution of IBD incidence with a second modest rise at 60–70 years (40). However, the second peak was not detected in this study. With the progression of global aging, IBD prevalence is on the rise among the elderly. A recent cohort study in Lothian (Scotland) has reported that IBD prevalence rates are notably high among people aged 60–79 years (1,178/100,000) or over 80 years (1,042/100,000) and should steadily increase in the next decade (41). Increasing IBD prevalence with age has also been observed in metropolitan Sydney (42). The higher prevalence of IBD among the elderly will put great pressure on healthcare systems because when receiving immunosuppressive therapies, older patients are more likely to develop opportunistic infections, sepsis, comorbidities, and malignancy (43).

By considering the population structure, our models have forecast the rising disease burden of IBD in China in the near future, and have predicted that China will transition to the Compounding Prevalence stage around 2030. The evolution of IBD can be stratified into four epidemiological stages: Emergence, Acceleration in Incidence, Compounding Prevalence, and Prevalence Equilibrium (44). Currently, newly industrialized countries such as China are in the Acceleration in Incidence stage, which is characterized by rapidly rising incidence and overall low prevalence (44). Since IBD is a chronic, incurable disease of the young, decades of high incidence and low mortality have caused Compounding Prevalence of IBD in the West, despite stabilizing or declining incidence (45). The phenomenon represents that in a lifelong disease like IBD, the prevalence will steadily accumulate and escalate as long as incidence surpasses mortality. According to our predictions, in China, IBD incidence rates are projected to rise further until a plateau in 2030, while the age-standardized death rates of IBD should remain low in the next 25 years. Thus, China is estimated to enter the Compounding Prevalence stage around 2030, echoing the experience of the West at the turn of the twenty-first century (7). Moreover, the number of IBD-related deaths is estimated to increase from 2020 to 2044, possibly due to aging patients with previously diagnosed IBD. China has one of the fastest-growing aging populations in the world. The population of people over 60 years old in China is projected to reach 28% (about 402 million) by 2040 as a result of longer life expectancy and declining fertility rates (46) (Supplementary Figure 2). The present study showed that the mortality of IBD increased exponentially with aging, and the prevalence rate of IBD reached a peak at around 60–70 years old (Figure 2). The large population size (over 1.4 billion), severe aging problem along with the climbing incidence together forecast a substantial disease burden in China in the coming years.

While there are shared pathophysiological and clinical features of IBD between China and western countries, the management of IBD in China is faced with distinct challenges, including limited medical resources, inadequate patient education, and the lack of standardized training programs for IBD specialists (27). There are currently only 63 referral centers for IBD in China, which fails to meet the sharp increase in IBD prevalence (27). In the last 5 years, the China Crohn's and Colitis Foundation (CCCF) has made great efforts to satisfy the rapidly growing need of IBD patients. Nearby certified IBD specialists can easily be found via the CCCF website or the WeChat (the most popular Chinese social media application) platform to facilitate early diagnosis and treatment. By December 2020, 371 CCCF specialists had been certified, covering 27 provinces and 178 hospitals, forming a comprehensive network of IBD specialists in China (27). Given the considerable heterogeneity of socioeconomic status across regions in China, it is essential for healthcare systems to prepare their infrastructure and personnel to provide an equitable, affordable, and accessible distribution of care for IBD patients in China, especially with the Compounding Prevalence challenges ahead (47).

Using the latest data from the GBD 2019, this study provided a comprehensive and long-term assessment of the burden of IBD in China. To the best of our knowledge, this is the first study that reported the predicted numbers and rates of incidence and mortality of IBD in China in the next 25 years. Besides these strengths, there are still several limitations. Firstly, we assessed the burden of IBD at the national level and did not include a detailed analysis among Chinese ethnic populations or across geographic regions. Population-based studies in the Asia-Pacific region have reported that within nine areas in China, IBD incidence rates vary from 0.54/100,000 in Xi'an to 3.64/100,000 in Guangzhou, which may be associated with the degree of urbanization (25). Secondly, the GBD data did not divide IBD into Crohn's disease and ulcerative colitis. In Asia, the incidence of ulcerative colitis is twice as much as that of Crohn's disease. However, the ratio has decreased over time because of increased awareness and advances in medical technology such as colonoscopy (26). Given the difference in epidemiological patterns, it is necessary to analyze these two IBD subtypes separately. Thirdly, our study is subject to general limitations of the GBD studies, such as the availability and quality of primary data, especially for non-fatal outcomes (11, 48, 49). Although preliminary data on the IBD burden in China may be sparse and not nationally representative, the GBD studies have applied sophisticated statistical modeling to fill the gap. However, fundamental improvements in the estimation accuracy still rely on optimized primary data collection, including complete coverage and preferred case definitions (11).

To conclude, China has experienced a rapid rise in IBD prevalence and incidence during the past three decades. Due to the improvements in health care, the fatal burden of IBD decreased, but the non-fatal burden steadily increased. The young age of onset, consistently low mortality, and high incidence rates should lead to the Compounding Prevalence of IBD in China in the near future. In addition to the expanded volume of patients, future gastroenterology clinics will also be challenged by an aging IBD population with complex comorbidities. Multiple strategies can be taken to mitigate the burden of IBD, including restriction of environmental exposures, innovations in healthcare delivery, pathogenesis research, and the identification of novel therapeutic targets (6). Over the next decades, the substantial burden of IBD in China calls for the collaboration of health policymakers, clinicians, and researchers.

## Data availability statement

The datasets presented in this study can be found in online repositories. The names of the repository/repositories and accession number(s) can be found in the article/Supplementary material.

## Ethics statement

The studies involving human participants were reviewed and approved by Institutional Review Board of Sir Run Run Shaw Hospital of Zhejiang University. Written informed consent to participate in this study was provided by the participants' legal guardian/next of kin.

## Author contributions

BS and QC contributed to the conception, design of the study, and revised it critically for important intellectual content. BS and WY contributed to the acquisition of data, analysis, and interpretation of data. WY drafted the article. All authors contributed to the article and approved the submitted version.

## Conflict of interest

The authors declare that the research was conducted in the absence of any commercial or financial relationships that could be construed as a potential conflict of interest.

## Publisher's note

All claims expressed in this article are solely those of the authors and do not necessarily represent those of their affiliated organizations, or those of the publisher, the editors and the reviewers. Any product that may be evaluated in this article, or claim that may be made by its manufacturer, is not guaranteed or endorsed by the publisher.

## Abbreviations

IBD: inflammatory bowel disease
GBD: Global Burden of Disease
YLDs: years of life lived with disability
YLLs: years of life lost
DALYs: disability-adjusted life years
GHDx: Global Health Data Exchange.
