# Corrigendum: Landscape and predictions of inflammatory bowel disease in China: China will enter the Compounding Prevalence stage around 2030

**DOI:** 10.3389/fpubh.2022.1083211

**Published:** 2022-11-30

**Authors:** Bule Shao, Wenjing Yang, Qian Cao

**Affiliations:** ^1^Department of Gastroenterology, Sir Run Run Shaw Hospital, Zhejiang University School of Medicine, Hangzhou, China; ^2^Zhejiang University-University of Edinburgh Institute, Zhejiang University School of Medicine, Hangzhou, China

**Keywords:** disease burden, China, prediction, public health, inflammatory bowel disease, age-period-cohort analysis

In the published article, there was an error in Figure 2 and Supplementary Figure 1 as published. The same typos when generated by the R software to PDF, i.e., the X-axis label “≥ 95” was incorrectly generated as “= 95”. Although the typos will not affect the results and conclusions of the manuscript, the authors would like to upload the correct version of the figures for better reading experiences for readers.

The authors apologize for these errors and state that this does not change the scientific conclusions of the article in any way. The original article has been updated.

## Publisher's note

All claims expressed in this article are solely those of the authors and do not necessarily represent those of their affiliated organizations, or those of the publisher, the editors and the reviewers. Any product that may be evaluated in this article, or claim that may be made by its manufacturer, is not guaranteed or endorsed by the publisher.

