# Supplementary material for: Landscape and predictions of inflammatory bowel disease in China: China will enter the Compounding Prevalence stage around 2030
Source: Front Public Health. 2022 Oct 25;10:1032679. doi: 10.3389/fpubh.2022.1032679 (PMC9641090; doi:10.3389/fpubh.2022.1032679)
Supplement: Supplementary file 1 [file Table_1.pdf]

Supplementary Table 1. IBD burden metrics by sex in China in 1990 and 2019 and the annual percentage change in age-standardized rates.

	1990				2019				1990-2019	
	Counts [$\times 10^3$ (95% UI)]		Age-standardized rate [per 100,000 (95% UI)]		Counts [$\times 10^3$ (95% UI)]		Age-standardized rate [per 100,000 (95% UI)]		Annual change rate [% (95% CI)]	
	Female	Male	Female	Male	Female	Male	Female	Male	Female	Male
**Prevalence**	107 (88.2, 127)	133 (110, 160)	20.7 (17.2, 24.5)	25.1 (20.9, 30.2)	427 (366, 498)	484 (411, 571)	44.3 (37.9, 51.4)	50.0 (42.5, 58.5)	1.14 (1.05, 1.26)	0.99 (0.90, 1.09)
**Incidence**	6.81 (5.70, 8.07)	10.4 (8.60, 12.3)	1.20 (1.02, 1.42)	1.72 (1.44, 2.05)	22.6 (19.4, 26.6)	28.9 (24.6, 33.9)	2.65 (2.29, 3.08)	3.35 (2.88, 3.88)	1.20 (1.12, 1.31)	0.95 (0.86, 1.04)
**Deaths**	3.00 (1.82, 3.97)	2.56 (1.59, 3.62)	0.84 (0.52, 1.18)	0.94 (0.58, 1.46)	2.14 (1.65, 2.62)	2.54 (1.88, 3.15)	0.24 (0.19, 0.30)	0.38 (0.30, 0.47)	-0.71 (-0.81, -0.51)	-0.59 (-0.76, -0.35)
**YLDs**	17.0 (11.1, 24.3)	19.9 (12.9, 28.0)	3.26 (2.12, 4.62)	3.74 (2.43, 5.24)	64.7 (42.5, 90.8)	71.2 (47.2, 100)	6.79 (4.47, 9.48)	7.38 (4.87, 10.3)	1.08 (0.96, 1.24)	0.98 (0.84, 1.13)
**YLLs**	106 (54.0, 144)	91.2 (49.0, 122)	22.2 (11.9, 29.5)	20.3 (12.0, 27.8)	39.4 (30.1, 48.3)	57.2 (41.1, 70.8)	4.77 (3.67, 5.80)	7.51 (5.66, 9.14)	-0.79 (-0.85, -0.56)	-0.63 (-0.75, -0.35)
**DALYs**	123 (69.6, 161)	111 (67.6, 143)	25.5 (14.9, 32.6)	24.1 (15.4, 31.6)	104 (79.7, 134)	128 (98.5, 162)	11.6 (8.99, 14.8)	14.9 (11.5, 18.5)	-0.55 (-0.67, -0.18)	-0.38 (-0.56, -0.048)

IBD, inflammatory bowel disease; 95% UI, 95% uncertainty interval; 95% CI, 95% confidence interval; YLLs, years of life lost; YLDs, years of life lived with disability; DALYs, disability-adjusted life-years.
