# Supplementary material for: Landscape and predictions of inflammatory bowel disease in China: China will enter the Compounding Prevalence stage around 2030
Source: Front Public Health. 2022 Oct 25;10:1032679. doi: 10.3389/fpubh.2022.1032679 (PMC9641090; doi:10.3389/fpubh.2022.1032679)
Supplement: Supplementary file 2 [file Table_2.pdf]

**Supplementary Table 2.** Age patterns by sex of IBD prevalence, incidence and deaths in China in 2019.

| Age (years) | Prevalence | | | | Incidence | | | | Deaths | | | |
|---|---|---|---|---|---|---|---|---|---|---|---|---|
| | Counts (95% UI) | | Rate [per 100,000 (95% UI)] | | Counts (95% UI) | | Rate [per 100,000 (95% UI)] | | Counts (95% UI) | | Rate [per 100,000 (95% UI)] | |
| | Female | Male | Female | Male | Female | Male | Female | Male | Female | Male | Female | Male |
| **1-4** | 9.13 (5.50, 13.6) | 12.3 (7.85, 17.8) | 0.030 (0.018, 0.044) | 0.034 (0.022, 0.050) | 9.63 (5.84, 14.3) | 13.0 (8.30, 18.8) | 0.031 (0.019, 0.047) | 0.036 (0.023, 0.053) | 41.6 (27.7, 57.5) | 55.1 (34.6, 80.0) | 0.14 (0.090, 0.19) | 0.15 (0.097, 0.22) |
| **5-9** | 162 (114, 221) | 232 (168, 313) | 0.49 (0.34, 0.66) | 0.59 (2.07, 3.60) | 77.8 (55.6, 105) | 113 (82.2, 152) | 0.23 (0.17, 0.31) | 0.29 (0.21, 0.39) | 4.09 (2.92, 5.45) | 12.1 (7.95, 16.4) | 0.012 (0.0088, 0.016) | 0.031 (0.020, 0.042) |
| **10-14** | 692 (513, 916) | 1058 (794, 1381) | 2.14 (1.59, 2.83) | 2.76 (2.07, 3.60) | 223 (165, 299) | 351 (263, 463) | 0.69 (0.51, 0.93) | 0.92 (0.69, 1.21) | 3.43 (2.46, 4.46) | 6.23 (4.30, 8.44) | 0.011 (0.0072, 0.014) | 0.016 (0.011, 0.022) |
| **15-19** | 2786 (2104, 3626) | 3333 (2556, 4281) | 7.95 (6.00, 10.3) | 8.31 (6.37, 10.7) | 525 (395, 682) | 828 (627, 1067) | 1.50 (1.13, 1.95) | 2.06 (1.56, 2.66) | 3.44 (2.53, 4.60) | 4.82 (3.30, 6.90) | 0.0098 (0.0072, 0.013) | 0.012 (0.0082, 0.017) |
| **20-24** | 6198 (4738, 7813) | 8280 (6508, 10369) | 15.9 (12.1, 20.0) | 19.4 (15.2, 24.2) | 1037 (804, 1322) | 1556 (1218, 1961) | 2.65 (2.06, 3.38) | 3.64 (2.85, 4.59) | 5.77 (4.01, 7.74) | 13.8 (9.10, 18.6) | 0.015 (0.010, 0.020) | 0.032 (0.021, 0.043) |
| **25-29** | 16081 (12581, 20411) | 19761 (15809, 24459) | 29.6 (23.1, 37.5) | 35.1 (28.1, 43.4) | 2045 (1568, 2568) | 2840 (2177, 3574) | 3.76 (2.88, 4.72) | 5.04 (3.86, 6.34) | 9.75 (6.40, 13.3) | 22.0 (14.6, 28.5) | 0.018 (0.012, 0.024) | 0.039 (0.026, 0.051) |
| **30-34** | 29710 (23315, 36291) | 34563 (27435, 41981) | 46.6 (36.5, 56.9) | 52.9 (42.0, 64.3) | 2910 (2192, 3669) | 3795 (2869, 4819) | 4.56 (3.44, 5.75) | 5.81 (4.39, 7.38) | 11.6 (7.53, 15.4) | 29.1 (19.0, 38.3) | 0.018 (0.012, 0.024) | 0.045 (0.029, 0.059) |
| **35-39** | 36641 (29897, 43650) | 36875 (29848, 44526) | 74.0 (60.4, 88.2) | 71.7 (58.1, 86.6) | 2455 (1873, 3101) | 3085 (2347, 3870) | 4.96 (3.78, 6.26) | 6.00 (4.57, 7.53) | 16.5 (11.1, 22.5) | 30.6 (20.8, 40.5) | 0.033 (0.022, 0.045) | 0.060 (0.040, 0.079) |
| **40-44** | 42539 (34988, 49931) | 45279 (36401, 54335) | 85.5 (70.3, 100) | 87.3 (70.2, 105) | 2426 (1880, 3075) | 3014 (2316, 3840) | 4.88 (3.78, 6.18) | 5.81 (4.46, 7.40) | 26.3 (16.8, 36.1) | 42.9 (29.0, 56.6) | 0.053 (0.034, 0.073) | 0.083 (0.056, 0.11) |
| **45-49** | 53495 | 62366 | 89.9 | 101 | 2743 | 3417 | 4.61 | 5.53 | 37.2 | 80.9 | 0.062 | 0.13 |

| | | | | | | | | | | | | |
|---|---|---|---|---|---|---|---|---|---|---|---|---|
| | (44612, 63399) | (50809, 75240) | (74.9, 106) | (82.2, 122) | (2086, 3567) | (2556, 4464) | (3.50, 5.99) | (4.13, 7.22) | (25.0, 49.7) | (53.3, 107.9) | (0.042, 0.083) | (0.086, 0.17) |
| **50-54** | 60570 (50389, 72849) | 70078 (57601, 84492) | 97.3 (80.9, 117) | 112 (91.7, 134) | 2590 (1970, 3372) | 3172 (2388, 4160) | 4.16 (3.16, 5.42) | 5.05 (3.80, 6.62) | 64.9 (41.1, 87.6) | 110 (72.0, 149) | 0.10 (0.066, 0.14) | 0.17 (0.11, 0.24) |
| **55-59** | 50093 (42476, 60433) | 57124 (47772, 69367) | 106 (90.0, 128) | 120 (100, 146) | 1754 (1313, 2303) | 2179 (1602, 2875) | 3.72 (2.78, 4.88) | 4.57 (3.36, 6.04) | 76.0 (49.6, 101) | 138 (86.7, 185) | 0.16 (0.10, 0.21) | 0.29 (0.18, 0.39) |
| **60-64** | 43095 (35983, 52167) | 49562 (41084, 61192) | 110 (92.0, 133) | 126 (104, 155) | 1295 (943, 1722) | 1642 (1186, 2209) | 3.31 (2.41, 4.41) | 4.16 (3.00, 5.60) | 109 (73.3, 141) | 178 (116, 230) | 0.28 (0.19, 0.36) | 0.45 (0.29, 0.58) |
| **65-69** | 38509 (32087, 46791) | 43188 (35363, 53853) | 107 (89.5, 130.6) | 125 (102, 156) | 1056 (768, 1445) | 1298 (934, 1806) | 2.95 (2.14, 4.03) | 3.76 (2.70, 5.23) | 161 (115, 212) | 249 (159, 327) | 0.45 (0.32, 0.59) | 0.72 (0.46, 0.95) |
| **70-74** | 23927 (19453, 29318) | 27743 (22276, 34434) | 97.5 (79.3, 120) | 119 (95.5, 148) | 649 (457, 902) | 800 (561, 1156) | 2.64 (1.87, 3.68) | 3.43 (2.40, 4.96) | 230 (170, 293) | 328 (228, 428) | 0.94 (0.69, 1.19) | 1.41 (0.98, 1.83) |
| **75-79** | 12523 (9460, 15641) | 14810 (11306, 18562) | 79.7 (60.2, 99.6) | 105 (80.0, 131) | 380 (263, 556) | 447 (300, 672) | 2.42 (1.68, 3.54) | 3.16 (2.12, 4.75) | 266 (199, 344) | 353 (242, 452) | 1.69 (1.26, 2.19) | 2.50 (1.71, 3.20) |
| **80-84** | 6410 (4585, 8317) | 7487 (5532, 9550) | 60.0 (42.9, 77.8) | 89.4 (114, 66.1) | 240 (166, 345) | 245 (165, 365) | 2.24 (1.55, 3.23) | 2.93 (1.97, 4.36) | 385 (289, 496) | 416 (309, 557) | 3.60 (2.71, 4.64) | 4.97 (3.69, 6.64) |
| **85-89** | 2456 (1738, 3356) | 2318 (3039, 1702) | 44.4 (31.4, 60.7) | 77.8 (57.1, 102) | 116 (80.1, 166) | 81.6 (53.9, 119) | 2.09 (1.45, 3.01) | 2.74 (1.81, 4.00) | 352 (264, 477) | 377 (296, 484) | 6.36 (4.77, 8.63) | 12.6 (9.94, 16.3) |
| **90-94** | 659 (476, 914) | 271 (196, 366) | 35.5 (25.6, 49.2) | 70.1 (50.6, 94.7) | 37.9 (25.5, 55.2) | 10.2 (6.81, 15.1) | 2.04 (1.38, 2.97) | 2.63 (1.76, 3.91) | 225 (166, 300) | 84.9 (67.5, 111) | 12.1 (8.92, 16.1) | 22.0 (17.5, 28.7) |
| **≥95** | 128 (86.0, 187) | 20.6 (13.9, 29.6) | 31.2 (20.9, 45.5) | 57.0 (38.3, 81.8) | 8.43 (5.41, 13.1) | 0.91 (0.58, 1.45) | 2.05 (1.32, 3.18) | 2.51 (1.59, 4.01) | 106 (75.1, 141) | 9.19 (6.51, 14.7) | 25.9 (18.3, 34.5) | 25.4 (18.0, 40.7) |

IBD, inflammatory bowel disease; 95% UI, 95% uncertainty interval.
