# Supplementary material for: Landscape and predictions of inflammatory bowel disease in China: China will enter the Compounding Prevalence stage around 2030
Source: Front Public Health. 2022 Oct 25;10:1032679. doi: 10.3389/fpubh.2022.1032679 (PMC9641090; doi:10.3389/fpubh.2022.1032679)
Supplement: Supplementary file 3 [file Table_3.pdf]

Supplementary Table 3. Age patterns by sex of IBD YLDs, YLLs and DALYs in China in 2019.

Age (years)	YLDs				YLLs				DALYs			
	Counts (95% UI)		Rate [per 100,000 (95% UI)]		Counts (95% UI)		Rate [per 100,000 (95% UI)]		Counts (95% UI)		Rate [per 100,000 (95% UI)]	
	Female	Male	Female	Male	Female	Male	Female	Male	Female	Male	Female	Male
**1-4**	1.45 (0.79, 2.48)	1.95 (1.08, 3.30)	0.0047 (0.0026, 0.0081)	0.0054 (0.0030, 0.0092)	3581 (2384, 4947)	4737 (2978, 6879)	11.7 (7.77, 16.1)	13.2 (8.32, 19.2)	3583 (2385, 4949)	4739 (2980, 6880)	11.7 (7.78, 16.1)	13.3 (8.33, 19.2)
**5-9**	25.7 (15.2, 41.6)	36.8 (21.9, 58.9)	0.077 (0.046, 0.12)	0.094 (0.056, 0.15)	335 (239, 446)	988 (649, 1344)	1.00 (0.72, 1.34)	2.52 (1.65, 3.42)	360 (263, 474)	1024 (692, 1379)	1.08 (0.79, 1.42)	2.61 (1.76, 3.51)
**10-14**	110 (65.0, 170)	168 (100, 256)	0.34 (0.20, 0.53)	0.44 (0.26, 0.67)	262 (188, 341)	475 (328, 645)	0.81 (0.58, 1.05)	1.24 (0.86, 1.68)	371 (288, 471)	643 (476, 829)	1.15 (0.89, 1.46)	1.68 (1.24, 2.16)
**15-19**	489 (293, 747)	528 (319, 799)	1.40 (0.84, 2.13)	1.32 (0.79, 1.99)	246 (180, 328)	344 (235, 492)	0.70 (0.51, 0.94)	0.86 (0.59, 1.23)	735 (525, 1009)	872 (625, 1184)	2.10 (1.50, 2.88)	2.17 (1.56, 2.95)
**20-24**	1041 (621, 1560)	1301 (750, 1973)	2.66 (1.59, 3.99)	3.04 (1.75, 4.61)	384 (267, 515)	919 (606, 1236)	0.98 (0.68, 1.32)	2.15 (1.42, 2.89)	1426 (984, 1965)	2221 (1581, 2932)	3.65 (2.52, 5.03)	5.19 (3.70, 6.85)
**25-29**	2709 (1667, 4023)	3060 (1819, 4525)	4.98 (3.07, 7.40)	5.43 (3.23, 8.03)	601 (394, 820)	1358 (897, 1754)	1.11 (0.73, 1.51)	2.41 (1.59, 3.11)	3310 (2211, 4611)	4418 (3128, 5938)	6.09 (4.07, 8.48)	7.84 (5.55, 10.5)
**30-34**	4900 (3006, 7276)	5311 (3218, 7986)	7.68 (4.71, 11.4)	8.13 (4.93, 12.2)	655 (426, 873)	1648 (1078, 2167)	1.03 (0.67, 1.37)	2.52 (1.65, 3.32)	5555 (3611, 7925)	6960 (4837, 9513)	8.71 (5.66, 12.4)	10.7 (7.41, 14.6)
**35-39**	6100 (3859, 8919)	5580 (3481, 8272)	12.3 (7.80, 18.0)	10.9 (6.77, 16.1)	853 (573, 1161)	1582 (1073, 2092)	1.72 (1.16, 2.35)	3.08 (2.09, 4.07)	6953 (4705, 9792)	7163 (4996, 9869)	14.0 (9.51, 19.8)	13.9 (9.72, 19.2)
**40-44**	6800 (4422, 9800)	6798 (4270, 9865)	13.7 (8.89, 19.7)	13.1 (8.23, 19.0)	1230 (784, 1688)	2007 (1356, 2647)	2.47 (1.57, 3.39)	3.87 (2.61, 5.10)	8029 (5615, 11227)	8805 (6253, 11863)	16.1 (11.3, 22.6)	17.0 (12.1, 22.9)
**45-49**	8088 (5245, 11702)	9285 (5913, 13635)	13.6 (8.81, 19.7)	15.0 (9.56, 22.1)	1556 (1048, 2080)	3389 (2232, 4521)	2.61 (1.76, 3.49)	5.48 (3.61, 7.31)	9644 (6613, 13269)	12674 (9064, 17361)	16.2 (11.1, 22.3)	20.5 (14.7, 28.1)
**50-54**	8926 (5659, 13194)	10333 (6556, 14907)	14.3 (9.09, 21.2)	16.4 (10.4, 23.7)	2407 (1524, 3249)	4081 (2671, 5538)	3.87 (2.45, 5.22)	6.49 (4.25, 8.81)	11333 (7932, 15751)	14413 (10223, 19413)	18.2 (12.7, 25.3)	22.9 (16.3, 30.9)

**55-59**	7310 (4690, 10408)	8345 (5446, 12073)	15.5 (9.94, 22.1)	17.5 (11.4, 25.3)	2464 (1606, 3265)	4461 (2811, 5989)	5.22 (3.40, 6.92)	9.36 (5.90, 12.6)	9774 (6955, 13075)	12806 (9432, 16848)	20.7 (14.7, 27.7)	26.9 (19.8, 35.4)
**60-64**	6191 (3950, 8988)	7115 (4536, 10337)	15.8 (10.1, 23.0)	18.0 (11.5, 26.2)	3017 (2037, 3921)	4951 (3224, 6398)	7.72 (5.21, 10.0)	12.5 (8.17, 16.2)	9208 (6770, 12232)	12067 (8926, 15470)	23.6 (17.3, 31.3)	30.6 (22.6, 39.2)
**65-69**	5472 (3444,8042)	6114 (3869, 8879)	15.3 (9.61, 22.4)	17.7 (11.2, 25.7)	3755 (2674, 4926)	5817 (3700, 7635)	10.5 (7.46, 13.7)	16.8 (10.7, 22.1)	9227 (6870, 12009)	11930 (8791, 15222)	25.7 (19.5, 33.5)	34.5 (25.4, 44.1)
**70-74**	3380 (2138, 4895)	3840 (2453, 5656)	13.8 (8.72, 20.0)	16.5 (10.5, 24.2)	4390 (3240, 5577)	6256 (4347, 8164)	17.9 (13.2, 22.7)	26.8 (18.6, 35.0)	7771 (6040, 9689)	10096 (7616, 12628)	31.7 (24.6, 39.5)	43.3 (32.6, 54.1)
**75-79**	1755 (1101, 2605)	2035 (1258, 3018)	11.2 (7.01, 16.6)	14.4 (8.90, 21.3)	3998 (2987, 5172)	5339 (3657, 6834)	25.5 (19.0, 32.9)	37.8 (25.9, 48.3)	5753 (4553, 7102)	7374 (5460, 9132)	36.6 (29.0, 45.2)	52.2 (38.6, 64.6)
**80-84**	902 (554, 1364)	1024 (639, 1509)	8.44 (5.19, 12.8)	12.2 (7.63, 18.0)	4454 (3347, 5737)	4825 (3579, 6445)	41.7 (31.3, 53.7)	57.6 (42.7, 77.0)	5356 (4181, 6638)	5849 (4584, 7459)	50.1 (39.1, 62.1)	69.8 (54.7, 89.1)
**85-89**	356 (213, 554)	315 (190, 470)	6.45 (3.85, 10.0)	10.6 (6.38, 15.8)	3106 (2329, 4210)	3351 (2632, 4304)	56.2 (42.1, 76.2)	113 (88.4, 145)	3463 (2681, 4558)	3666 (2917, 4624)	62.6 (48.5, 82.5)	123 (98.0, 155)
**90-94**	98.3 (58.6, 159)	37.2 (22.1, 59.1)	5.29 (3.15, 8.56)	9.63 (5.72, 15.3)	1542 (1134, 2047)	594 (472, 776)	83.0 (61.0, 110)	154 (122, 201)	1641 (1226, 2148)	631 (506, 813)	88.3 (66.0, 116)	163 (131, 210)
**≥95**	19.2 (11.0, 32.8)	2.85 (1.67, 4.68)	4.68 (2.69, 7.98)	7.88 (4.62, 12.9)	552 (389, 733)	48.5 (34.6, 75.8)	134 (94.8, 179)	134 (95.7, 210)	571 (412, 755)	51.3 (37.6, 78.5)	139 (101, 184)	142 (104, 217)

IBD, inflammatory bowel disease; YLLs, years of life lost; YLDs, years of life lived with disability; DALYs, disability-adjusted life-years; 95% UI, 95% uncertainty interval.
