# Supplementary figures and images for: Landscape and predictions of inflammatory bowel disease in China: China will enter the Compounding Prevalence stage around 2030
Source: Front Public Health. 2022 Oct 25;10:1032679. doi: 10.3389/fpubh.2022.1032679 (PMC9641090; doi:10.3389/fpubh.2022.1032679)

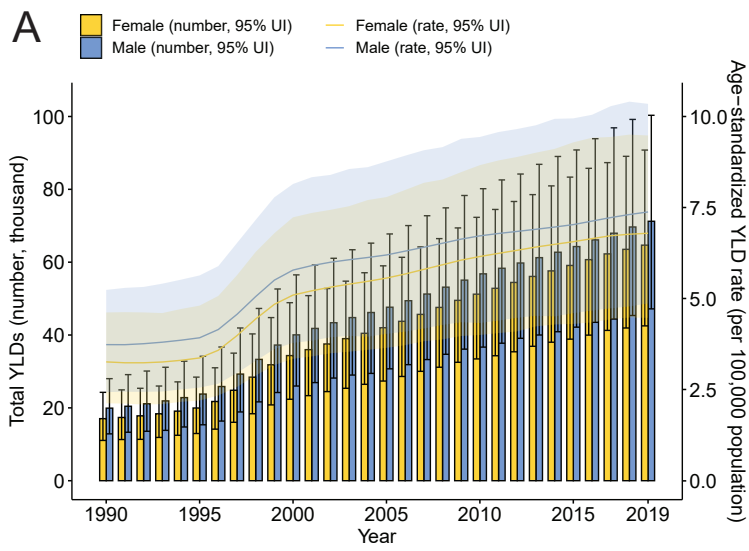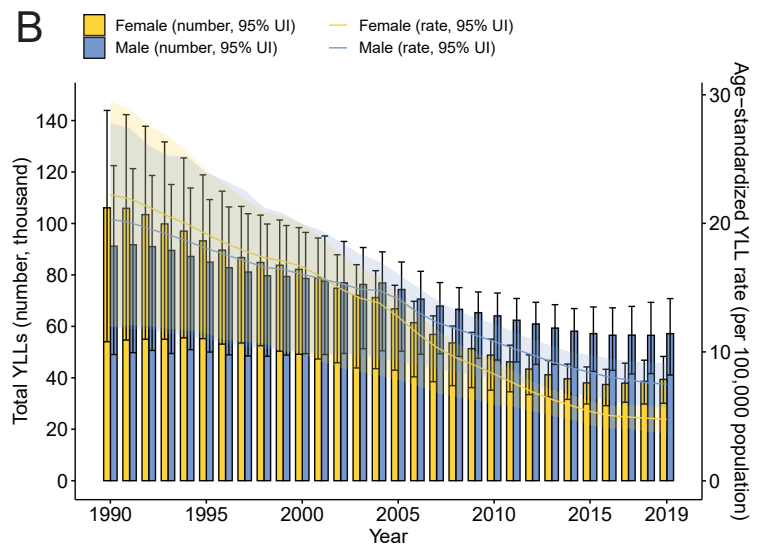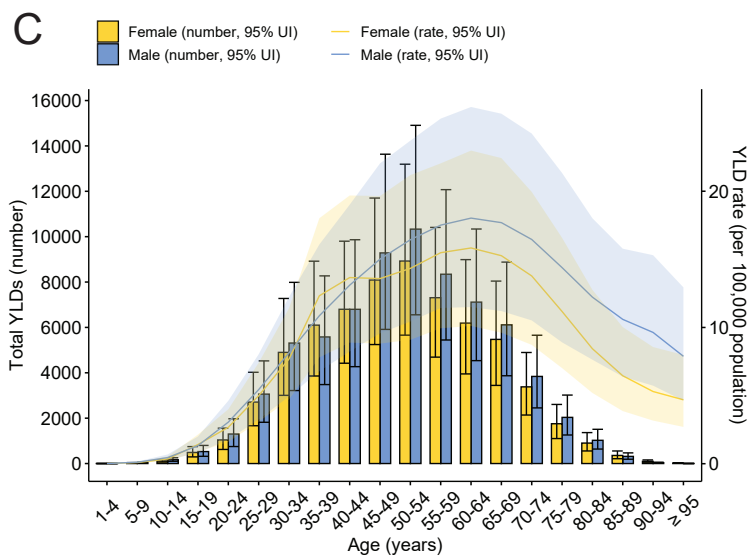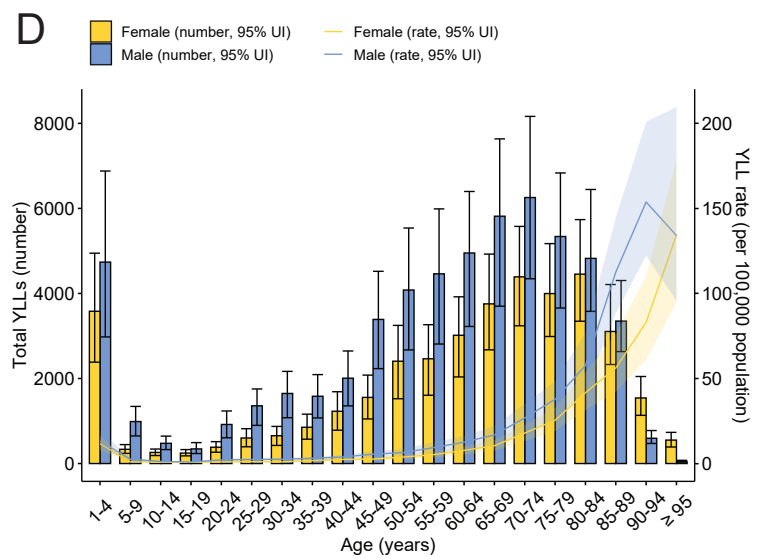

Supplement: Supplementary file 4 [file Image_1.pdf]

1990

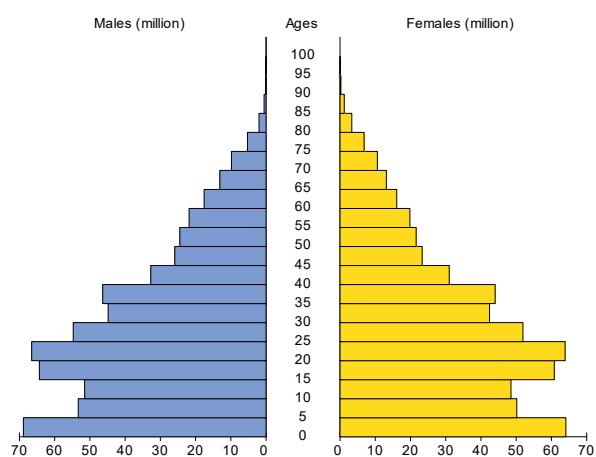

2000

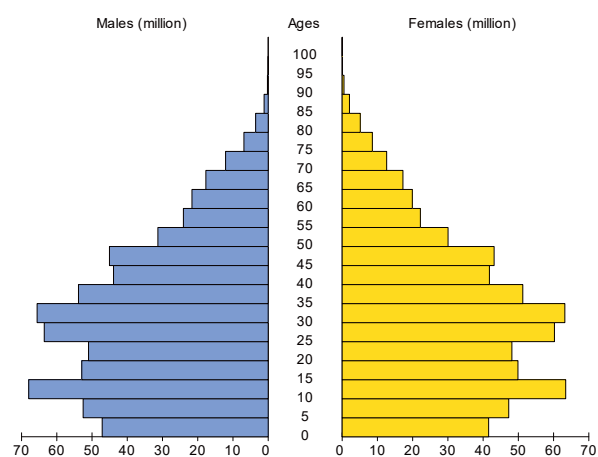

2020

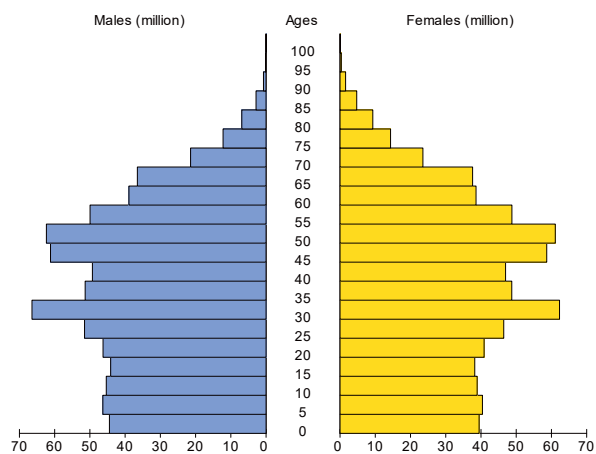

2040

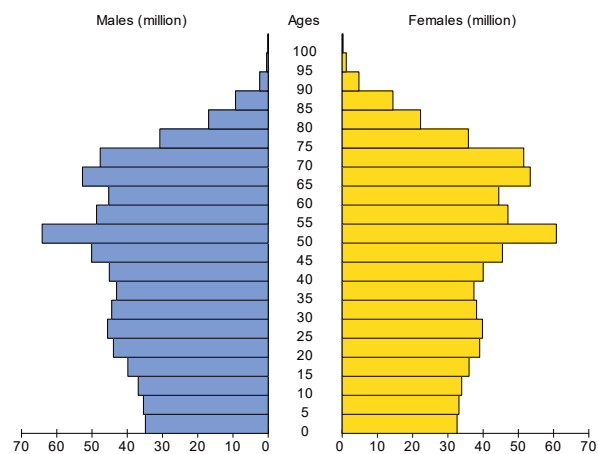

**Supplementary Figure 2.** Population pyramid of China from 1990 to 2040.

Supplement: Supplementary file 5 [file Image_2.pdf]
